# Network meta-analysis: users’ guide for pediatricians

**DOI:** 10.1186/s12887-018-1132-9

**Published:** 2018-05-29

**Authors:** Reem Al Khalifah, Ivan D. Florez, Gordon Guyatt, Lehana Thabane

**Affiliations:** 1 Department of Clinical Epidemiology & Biostatistics, McMaster University, Hamilton, ON Canada; 2 Department of Pediatrics, Division of Pediatric Endocrinology and Metabolism King Saud University, Riyadh, Saudi Arabia; 3 Department of Pediatrics, Universidad de Antioquia, Medellín, Colombia; 4 Department of Medicine, McMaster University, Hamilton, Canada; 5 Department of Pediatrics and Anesthesia, McMaster University, Hamilton, ON Canada

**Keywords:** Network meta-analysis, Multiple treatment comparisons, Multiple-treatment meta-analysis evidence synthesis, Evidence credibility, Evidence certainty, Pediatric

## Abstract

**Background:**

Network meta-analysis (NMA) is a powerful analytic tool that allows simultaneous comparison between several management/treatment alternatives even when direct comparisons of the alternatives (such as the case in which treatments are compared against placebo and have not been compared against each other) are unavailable. Though there are still a limited number of pediatric NMAs published, the rapid increase in NMAs in other areas suggests pediatricians will soon be frequently facing this new form of evidence summary.

**Discussion:**

Evaluating the NMA evidence requires serial judgments on the creditability of the process of NMA conduct, and evidence quality assessment. First clinicians need to evaluate the basic standards applicable to any meta-analysis (e.g. comprehensive search, duplicate assessment of eligibility, risk of bias, and data abstraction). Then evaluate specific issues related to NMA including precision, transitivity, coherence, and rankings.

**Conclusions:**

In this article we discuss how clinicians can evaluate the credibility of NMA methods, and how they can make judgments regarding the quality (certainty) of the evidence. We illustrate the concepts using recent pediatric NMA publications.

## Background

Randomized control trials (RCTs) constitute the optimal methodology to determine the effectiveness of medical interventions. When results against placebo or standard care suggest benefits outweigh harms, clinicians, patients and families must choose among several interventions. Making this choice optimally requires access to systematic summaries of the best available evidence.

For decades, investigators have provided these evidence summaries using systematic reviews and meta-analyses. By combining across studies, meta-analyses increase the precision of the effect estimate [1]. Conventional meta-analyses, however, address only single paired comparisons and are therefore of limited use when multiple reasonable options exist. One could envision a series of conventional meta-analyses addressing each possible paired comparison, but these have two major limitations. First, for the clinician or patient consumer, making sense of multiple meta-analyses would be challenging. Second, it is extremely likely that many of the possible paired comparisons will not have direct comparisons available; in such instances, there will be no conventional meta-analysis to consider.

Network meta-analysis (NMA), also known as multiple-treatment comparisons or multiple-treatment meta-analysis, provides a methodology to address this dilemma, taking advantage of two statistical innovations: the first is use of indirect comparisons—we can estimate the effect of A-B indirectly if both A and B have been compared against C (see next section). The second is that NMA statistical methods combining direct and indirect comparisons allow estimates of the relative effect of every alternative versus every other alternative.

Although the majority of published NMAs summarize evidence from RCTs, NMA of cohort studies – most often addressing the evidence regarding adverse events - are increasing [2, 3]. Moreover, given the recent development of the required methods, diagnostic test accuracy NMA may soon be available [4].

The first NMA addressing a pediatric issue evaluated the effects of indomethacin, ibuprofen, and placebo on patent ductus arteriosus closure in preterm infants [5]. Since then, the number of pediatric NMAs has increased [6–23] and, given development in other fields, one can anticipate a substantial further increase. This increase might, however, occur at a slower rate in the pediatric field because of the smaller number of RCTs relative to the adult literature.

The goal of this paper is to provide a users’ guide for pediatricians considering the application of the results of NMA addressing a therapeutic issue to their practice. Nonetheless, a minimum knowledge on Conventional meta-analysis is needed to understand most of the important concepts of NMA [24]. First, we introduce the reader to NMAs and provide criteria for evaluating the credibility of the NMA method. We then discuss the quality of the evidence (synonyms: certainty or confidence in evidence) obtained from a NMA (the NMA may have used optimal methods, but limitations of the underlying studies may still result in low quality evidence). To illustrate the processes of interpretation and implementation in the context of pediatric literature, we will present an example of the effects of 16 different mechanical ventilation modes on mortality among preterm infants with respiratory distress syndrome (RDS) [9], in addition to other examples from the pediatric literature when we could not illustrate the presented concepts using the mechanical ventilation NMA.

## Discussion

### Indirect evidence

Let us suppose that we are interested in the relative merits of two treatments, **A** and **B**. It may be that no study has directly compared the two treatments. If, however, investigators have compared both **A** and **B** against the same third alternative **C**, we can infer the relative effect of **A-B**. We do so by comparing the effect of **A-C** and **B-C** (the indirect comparison, Fig. 1.1).

**Fig. 1:**
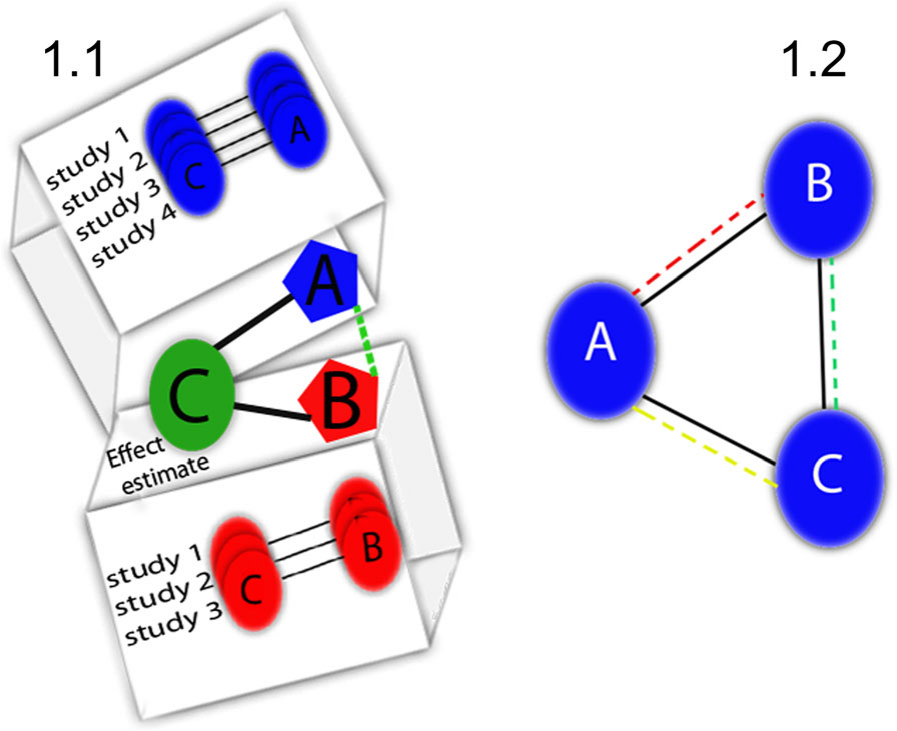
The concept of network meta-analysis. Each node (circle) is considered an intervention (A, B or C), sold lines represent loops of pairwise comparison (direct evidence), and doted lines represent loops of indirect comparison (indirect evidence). Indirect comparisons can be made via deduction from the common comparator. **1.1**. Indirect evidence of **A** versus **B** inferred from direct estimates of **A** versus **C** and **B** versus **C** Four studies formed the effect estimate for **A**-**C**, and 3 studies formed the effect estimate for **C**-**B**. The effect estimate of **A**-**B** was obtained from indirect evidence. **1.2**. Closed network shows the a closed network meta-analysis in a hypothetical example where all interventions were compared in RCT’s, therefore; direct and indirect evidence is available for all comparisons

For instance, if the relative risk (RR) of death in **A-C** is 0.5 (**A** reduces deaths relative to **C** by 50%) the RR of death in **B-C** is 1.0 (**B** has no effect on deaths relative to **C**), then it would be reasonable to infer that **A** will reduce death relative to **B** by 50%. Furthermore, if investigators have conducted both *direct* and *indirect* comparisons, we can combine the two and produce a *mixed* or *network estimate* (Fig. 1.2).

### Network meta-analysis

**Fig. 2:**
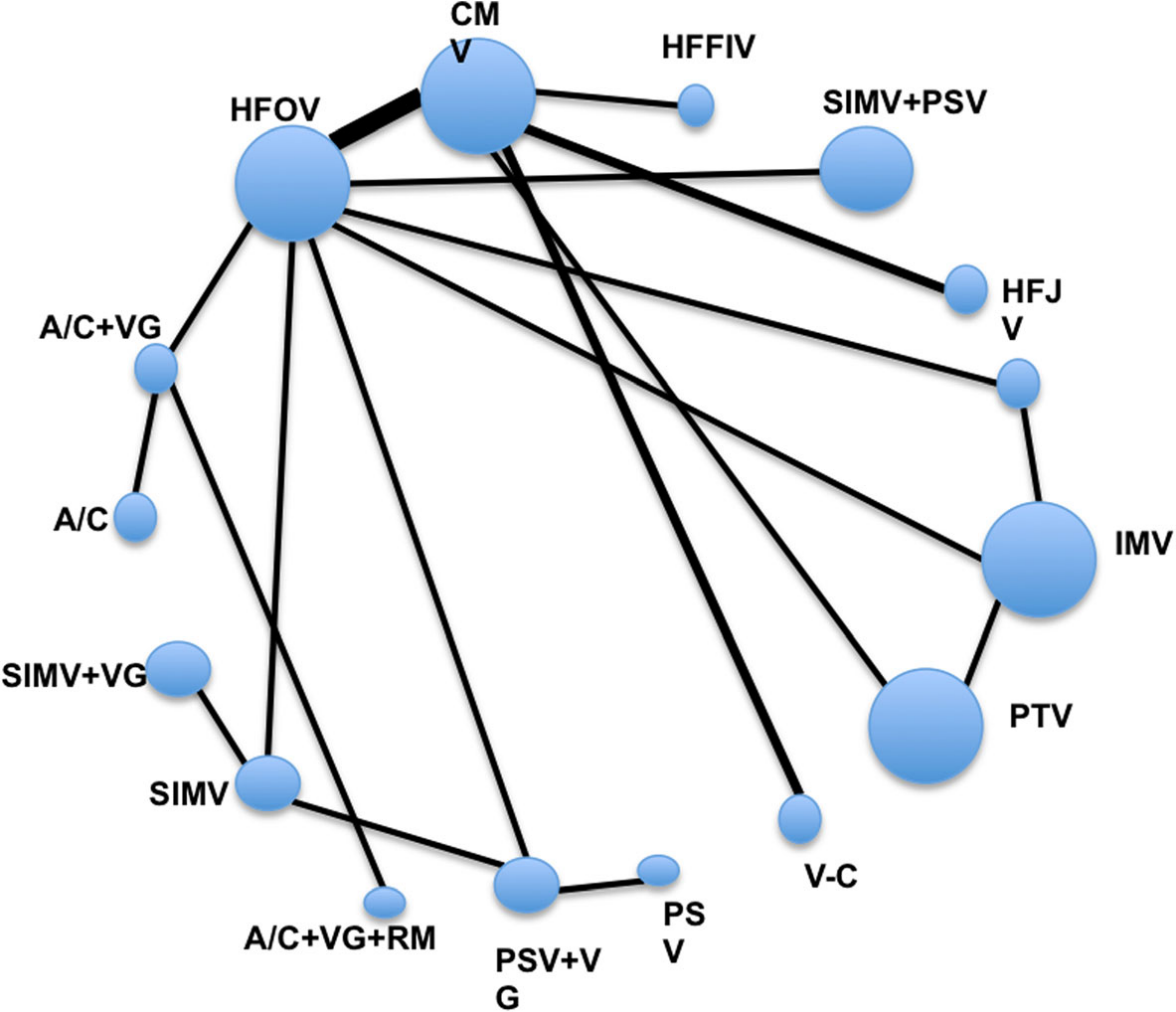
The geometry of the mechanical ventilation for premature infants NMA. A/C, assist-control ventilation; VG, volume guarantee ventilation; RM, recruitment maneuver; CMV, continuous mandatory ventilation; HFFIV, high-frequency flow interrupted ventilation; HFJV, high-frequency-jet ventilation; HFOV, high-frequency oscillatory ventilation; IMV, intermittent mandatory ventilation; PSV, pressure support ventilation; PTV, patient-triggered ventilation; SIMV, synchronized intermittent mechanical ventilation; SIPPV, synchronized intermittent positive pressure ventilation; V-C, volume-controlled. Wang C et al. Mechanical ventilation modes for respiratory distress syndrome in infants: a systematic review and network meta-analysis. Critical care (London, England). 2015, reprinted by permission of the publisher [9]

Ideally, an NMA will depict the available direct evidence in a figure; we refer to as a *network graph*. The circles (nodes) represent each intervention, and the lines between the nodes (called edges) represent head-to-head comparisons (Fig. 2) [25]. Some network graphs use the size of the nodes and the width of the edges to convey information about the amount of information available (circles convey the sample size of studies of a particular intervention and edges the number, sample size, or variance associated with the related direct comparisons, i.e. large node means larger sample size, and thick edge means increased number of studies included).

In comparison to conventional meta-analysis that relies exclusively on direct evidence, the NMA provides estimates of relative effectiveness among all interventions being compared, increases precision around effect estimates, ranks treatments, and enhances generalizability [26–28].

### Credibility of NMA methods

The conduct of NMA should adhere to standards of a traditional systematic review. Like a conventional meta-analysis, a credible NMA requires explicit eligibility criteria, comprehensive search, and assessment of evidence quality (Table 1).

**Table 1:** Guide for appraising NMA evidence

Credibility	Did the review explicitly address sensible question? Was the search for studies and selection comprehensive? Did the review assess evidence certainty? Did the review present results for the reader?
Certainty	What is the risk of bias of included studies? Were the results precise? Were results consistent across studies? How trustworthy are the indirect comparisons? Were results consistent between direct and indirect comparisons? Is there evidence for publication bias? Were treatment ranks presented and were they trustworthy?
Applicability	What is the overall quality of the evidence? What are the limitations of the evidence? Can I apply the results to my patients?

#### Did the review explicitly address a sensible question?

A well-formulated clinical question will typically follow the PICO format (P: population, I: intervention, C: comparator, O: outcomes) [29]. NMA uses the same format except that “I” and “C” (intervention and comparisons), include all the interventions compared against each other. Successful definitions of each element of the PICO are required to determine the studies eligible for the review and develop a priori hypotheses to address possible heterogeneity.

Although, the scope of the research question can vary from narrow to broad, it is essential that for any paired comparison within the NMA, it is plausible that we will, for each outcome of interest, observe similar effects across all patient populations being addressed [30, 31]. Eligibility criteria can be wide enough to permit the possibility of differences in effect across the included patients, interventions, and outcomes. For instance, effects may differ – among eligible studies- in more or less severely affected patients; across high and low doses and across shorter and longer follow-up.

An NMA that assessed the efficacy of asthma treatments strategies included all children with chronic asthma [12]. The definition of chronic asthma was not based on the Global Initiative for Asthma (GINA) asthma guidelines staging [32], nor did the authors present data on disease severity, or attempt subgroup analyses. The broad inclusion criteria and lack of subgroup hypotheses fail to address the differences in disease severity that might lead to differences in treatment response [33].

Another example relates to differences in the measurement of outcome [27, 34]. Two systematic reviews in asthma began with the goal of conducting an NMA; only one was successful. The first study evaluated the effectiveness of the various inhalation regimens on FEV1 improvement [18]. The systematic review revealed large variations in the way the 23 trials measured and reported FEV1. This heterogeneity prevented the review team from performing an NMA. The second NMA assessed the efficacy of treatments on reducing exacerbation [12]. Severe exacerbation was defined as patients needing hospital admission, a visit to the emergency department or a standard course of systemic corticosteroids. In this case, outcomes were reported similarly across trials and the authors presented pooled estimates.

#### Was the search for studies and selection comprehensive?

A comprehensive systematic search that identifies all pertinent available studies minimizes the risk of spurious findings from unrepresentative selection of studies. Since many reviews articles have demonstrated the inadequacy of searching only one database [35–37], an optimal search include all relevant electronic databases (e.g., Medline, Embase, Psycinfo, CENTRAL, CINAHL) [38]. Ideally, a search of the grey literature will minimize the risk of publication bias.

Subsequently, the team selects eligible studies [38]. The report should provide evidence of the reproducibility of assessment of study eligibility through review by at least 2 independent assessors, and present a figure summarizing each selection step in the eligibility determination process (identification of titles and abstracts; culling of titles and abstracts; review of full texts; final determination of eligibility) [39].

#### Did the review assess evidence certainty?

Certainty in effect estimates represents how trustworthy are the results and their conclusions [31]. Within any network, it is likely that the quality of the evidence differs across paired comparisons: high quality evidence may reveal that one treatment is superior to another, whereas we may have only low quality evidence regarding the relative merit of other treatments.

Making that rating requires a sequence of judgments relying on assessments of the quality of the direct and indirect evidence. Three articles were published on 2014 by the Grades of Recommendation, Assessment, Development and Evaluation (GRADE) working group, the Cochrane Collaboration, and the ISPOR-AMCP-NPC good practice task force [30, 31, 40] that extend quality of evidence assessment of meta-analysis to NMA. Following the GRADE approach, the overall confidence starts as high for direct, indirect, and network estimates that are derived from RCTs [31]. The evidence can be rated down from high to moderate, low, or very low quality based on the presence and magnitude of any of the 5 domains: Risk of bias (RoB), indirectness, imprecision, inconsistency, and publication bias [31].

Many prior published NMAs have not explicitly addressed all the recommended elements. Fortunately, however, some present the information required for a reader to make the necessary judgments. If the information is not available, then the credibility of the NMA is compromised [31].

Consider, for instance, the GRADE profile for the direct evidence of an NMA of antidepressant medications for improving depression symptoms in children (Table 2) [41]. The evidence certainty for Fluoxetine versus placebo was rated as very low as a result of high RoB, imprecision, and inconsistency. The Imipramine versus placebo comparison was rated as moderate, the only concern being imprecision. With this variation in evidence certainty, making sense of the results requires ratings of evidence quality for each pairwise comparison.

**Table 2:** GRADE evidence profile showing differences in the evidence certainty among two direct evidence comparisons in the depression treatment NMA for depression symptoms

Quality assessment				Quality			
№ of studies	Risk of bias	Inconsistency	Indirectness	Imprecision	Other considerations	Absolute Effect (95% CI)	
Fluoxetine vs. placebo							
8	Serious^a^	Serious^b^	Not serious	Serious^c^	None	SMD 0.26 SD lower (0.5 lower to 0.03 lower)	⊕OOO VERY LOW
Imipramine vs. placebo							
2	Not serious	Not serious	Not serious	Serious^d^	None	SMD 0 SD (0.27 lower to 0.26 higher)	⊕⊕⊕O MODERATE

*RCT* Randomised trials; *CI* Confidence interval, *SMD* Standardised mean difference [41]

^a^Selective outcome reporting, and incomplete outcome data

^b^Moderate heterogeneity I^2^ = 67.4%

^c^Upper CI very close to no effect

^d^SMD includes no effect

#### How do NMAs conduct analyses and present results?

There are two statistical approaches to perform NMA: a frequentist and a Bayesian approach [41, 42]. The frequentist approach is what clinicians will generally see in individual RCTs and conventional meta-analyses. The additional major aspect of Bayesian approaches is the specification of prior probabilities of treatment effects before beginning the data analysis and combining these priors and their precision with the estimate from the data to produce a posterior probability and its credible interval. Results in NMA are presented as effect estimates, typically odds ratios (ORs), or RR, hazard ratio (HR), mean difference (MD) with their 95% confidence interval (CI) (frequentist approach) or credible interval (CrI) (Bayesian approach), both of which describe the range of plausible truth around the point estimate.

Ideally, NMAs will present direct, indirect, and network estimates for each paired comparison. When, however, there are large numbers of comparisons, this becomes a challenging task. For example, the mechanical ventilation modes for RDS in preterm infants NMA included 16 different ventilation modes, yielding 120 comparisons- this probably requires an online appendix [9]. Ways to deal with this profusion of comparisons is to present effect estimates in a league table (all possible pairwise interventions compared to each other by cross-matching the interventions on the raw with those in the column), forest plots (all pairwise interventions compared to one reference intervention, or to the least efficacious intervention such as placebo), or evidence comparisons (direct, indirect, and NMA) for each intervention compared to one reference [13, 41, 43, 44].

### Certainty of NMA evidence

#### What is the risk of bias of included studies?

RoB conveys the likelihood that limitations in design or conduct of studies will result in estimates of treatment effect that vary systematically from the truth. The greater the RoB, the more appropriate it becomes to rate down the quality of the evidence [45, 46].

For assessing the RoB, authors may use an instrument such as the Cochrane RoB tool for RCTs [38]. This instrument assesses six elements: randomization sequence generation, concealment of allocation, blinding of participants, personnel and outcome assessors, completeness of follow-up, selective outcome reporting, and presence of other biases.

In the NMA of strategies for preventing asthma exacerbations, the authors used the Cochrane instrument to assess RoB [12] and judged all trials to be at low RoB. Although the authors did not provide an overall RoB judgment per comparison, it is possible -although tedious- for the pediatrician to make this rating if the NMA authors have presented ratings of RoB for each study in a table or figure. In this case, it is not a problem: since all studies were at low RoB, there is no need to rate down for RoB for any comparison.

#### Were the results precise?

The lack of adequate power to inform a particular outcome leads to imprecision [47]. One standard for assessing precision is to consider whether differences between intervention and control exclude chance (i.e. statistically significant). This has two limitations: first results may exclude no effect, but may not exclude an effect too small to be important; second, using this criterion, one would always rate down for precision if results were not statistically significant, no matter how narrow the CI or CrI.

Therefore, we suggest an alternative standard. To assess imprecision, one can consider whether decisions regarding choice of therapy will differ if the upper and lower CI or CrI represents the truth. Another way of thinking about this approach is to consider whether the CI or CrI excludes a minimally important difference (MID). The MID is a measure of the smallest change in the value of a patient-reported outcome, typically applied to outcomes such as quality of life measures [48].

For example, in the NMA of ventilation modes for infants, the comparison of synchronized-intermittent mechanical ventilation with volume-guarantee (SIMV+VG) versus high-frequency-jet-ventilation (HFJV), the point estimate suggests that SIMV+VG reduced mortality (HR = 0.23) [9]. However, the 95%CrI ranged from an extremely large reduction in mortality (HR = 0.03, reduction in hazard by 97%) to an almost doubling of hazard (HR = 1.46). Since the treatment choice will be different at each CrI end, the evidence quality is reduced for imprecision.

On the other hand, for the comparison SIMV+VG versus SIMV with pressure-support ventilation (SIMV+PSV), mortality was lower with SIMV+VG (HR = 0.12; 95%CrI 0.01, 0.86). Here, even the upper suggests a 14% reduction in hazard with SIMV+VG. Therefore, in this instance, there is no need to rate down the quality of the evidence for imprecision. Although, the width of the CrI may still be considered large and thus could be considered imprecise for outcomes such as hospital length of stay, any but the smallest reduction in mortality is critical. The judgment of importance is critically dependent on the absolute difference, in this case the absolute mortality risk difference: for instance, for 27 weeks infants with baseline mortality risk of 10%, the absolute mortality risk reduction with SIMV+VG versus SIMV+PSV would approximately be 9% if the point estimate of the HR (0.12) were accurate, and approximately 1.4% if the upper boundary of the CrI (0.86) represented the truth. The magnitude of the absolute difference is greater for even younger infants with higher mortality, and less for older infants with lower mortality (Table 3) [49–51].

**Table 3:** Anticipated absolute mortality among premature infants using SIMV+VG versus SIMV+PSV

	Relative effect Hazard ratio (95% CrI)	Anticipated absolute effects	
		Mortality risk with regular care	Mortality risk difference with SIMV+VG
GA > 30 weeks	0.12 (0.01 to 0.86)	5 per 100	4 fewer per 100 (5 fewer to 1 fewer)
GA 27–30 weeks	0.12 (0.01 to 0.86)	10 per 100	9 fewer per 100 (10 fewer to 1 fewer)
GA 25–26 weeks	0.12 (0.01 to 0.86)	50 per 100	42 fewer per 100 (49 fewer to 5 fewer)

The relative effect of SIMV+VG (and its 95% CrI) is based on the NMA estimates [9]; the absolute effect (and its 95% CI) is based on the assumed risk in the comparison group; mortality estimates with regular care are based on previous literature [49–51]

*GA* gestational age

In a complementary approach, authors can, for each direct comparison, assess imprecision by calculating the optimal information size (OIS), the number of patients or events needed for adequately powered individual study to avoid spurious findings [47]. This, however, ignores the contribution of the indirect comparisons to the network estimate. Methods to incorporate indirect estimates of OIS to NMA are under development [26].

### Were results consistent across studies?

One can expect variation between treatment effects –we call such variation “heterogeneity”. Heterogeneity can result from chance, or from differences in patients, interventions, comparisons, outcomes and methodology between studies (Table 4).

**Table 4:** Possible effect modifiers that may contribute to between study variability

Pure chance	
Different Risk of Bias Studies with high RoB might show large effect than those with low RoB.	
Different study Population: Baseline risk like gender, age (e.g., in some interventions, the effect could be larger in infants than in adolescents). Disease severity (e.g., in children with severe diseases the effect of x intervention might be smaller than in case of patients with mild disease). Treatment setting (e.g., patient with asthma enrolled from the emergency room will have different characteristics than those enrolled from the outpatient clinic).	
Different Interventions: Dose (larger doses are expected to be associated with larger effect ad sometimes with larger effect in terms of side effects). Route (intravenous administration may have larger effect if oral administration is impacted by absorption or hepatic metabolism). Duration (using the medication for longer duration may be associated with larger effect compared to shorter duration).	
Different comparators: Different standards of care when the standard of care is the comparator (e.g., in a diarrhea study, oral rehydration solution (ORS) is given to the control group in study A vs. ORS+ zinc supplement given to the control group in study B).	
Different ways in Outcome assessment: Definition (e.g., if fever is defined as 38.0 C in study A vs. 39.0 C in study B, this may result in diagnosing more patients with the fever in the study A). Measurement (e.g., if fever is measured using rectal temperature, compared to axillary temperature in another study; or standard methods in one study compared to non standard way).	

Assessing the degree of inconsistency in direct comparisons involves inspecting the point estimates and the degree of confidence or credible intervals overlap of each study in a forest plot. Two methods for formal statistical testing can complement visual inspection of forest plots – the test for heterogeneity (Cochran’s Q-test), and I^2^ (which quantifies the proportion of the total heterogeneity that is attributable to differences between the studies and ranges from 0 to 100%) [38].

**Fig. 3:**
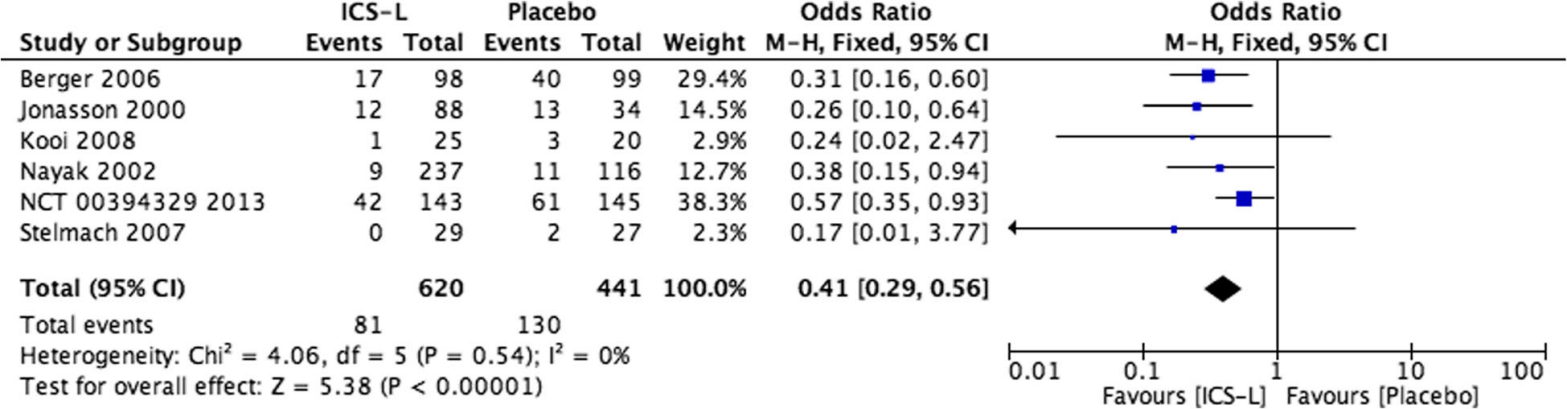
Forest plot comparing ICS-L vs. placebo for moderate or severe asthma exacerbations. Visual assessment indicates low heterogeneity, similar point estimates, overlapped CI, and I^2^ = 0 [12]. Zhao Y, et al. Effectiveness of drug treatment strategies to prevent asthma exacerbations and increase symptom-free days in asthmatic children: a network meta-analysis. The Journal of asthma: official journal of the Association for the Care of Asthma. 2015, reprinted by permission of the publisher (Taylor & Francis Ltd., WWW.tandfonline.com) [12]

For example, in the chronic asthma NMA, the authors presented direct comparison between low-dose inhaled corticosteroids (ICS-L) and placebo for moderate or severe exacerbation. Six trials contributed to the pooled estimate OR = 0.41 (95%CrI 0.29, 0.56). The forest plot shows similar point estimates, and CIs overlapped across all trials. The *P*-value for heterogeneity assessment was 0.54 (not significant), and I^2^ = 0% (Fig. 3), indicating a high level of consistency between results. Conversely, if there is substantial heterogeneity that is unexplained by subgroup analysis or meta-regression, we lose confidence in treatment effects and, in the GRADE approach, rate down the quality of evidence for inconsistency [31, 34, 52].

### How trustworthy are the indirect comparisons?

**Fig. 4:**
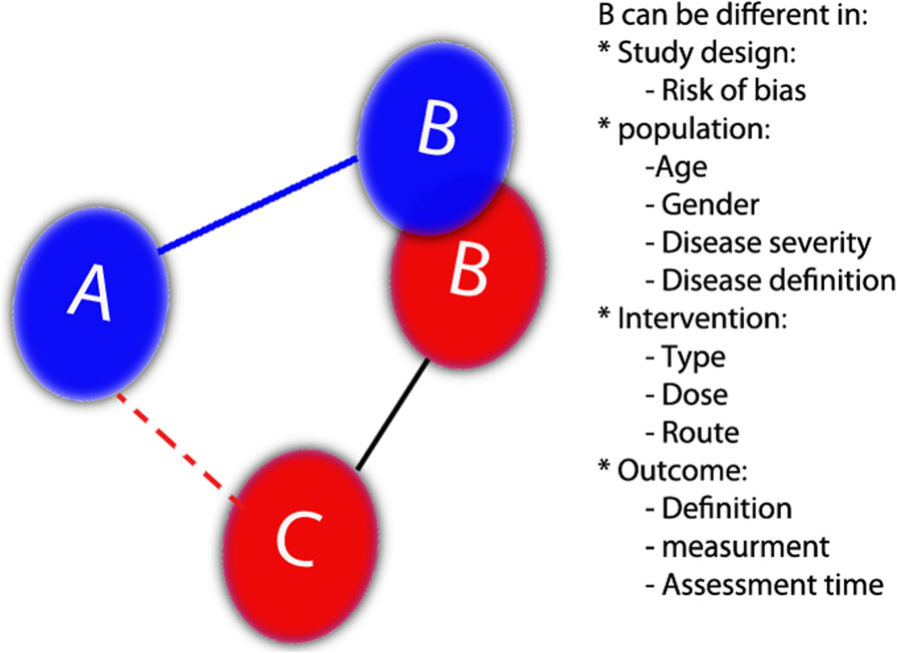
The diagram shows the concept of *intransitivity*. The doted line **A**–**C** shows the indirect evidence were inferences are being made. **B** is not shown as a unique intervention, rather as two different ways of **B** (Blue and Red). Intransitivity can occur when the distribution of a possible effect modifier is different between two groups

Trustworthiness of indirect comparisons - for instance, inferring the relative effect of A-B from A-C and B-C comparisons -requires similarity of patient population, comparators, outcomes, RoB, and optimal administration of the interventions under consideration (Fig. 4). In other words, A and B must both be optimally administered; the A-C and B-C comparisons must include similar patients; C must be similar; outcomes must be measured similarly; and studies would ideally be at low RoB. We refer to situations when this is not the case as “intransitivity”. Intransitivity reduces confidence in the results of indirect comparisons.

To illustrate the concept of intransitivity consider an NMA of comparative efficacy of psychotherapies for depression in children [10]. The comparison of interest is cognitive behavioral therapy (CBT) versus Problem-solving therapy (PST). We wish to make inferences regarding the effects of CBT versus PST from an indirect comparison: studies have compared both CBT and PST to wait list (WL) controls. The 14 RCTs comparing CBT versus WL used 8 different instruments to define depression; the 3 RCTs comparing PST versus WL (Table 5) used 2 of the 8, and a ninth that was not used at all in the CEB versus WL studies. Use of the different instruments could create differences in depression severity in the population that in turn could influence the magnitude of the treatment effect, suggesting possible intransitivity and consideration of consequent rating down of quality.

**Table 5:** Depression definition used in the psychotherapies NMA in the wait list (the common comparator) to illustrate the concept of intransitivity in the indirect evidence

Pairwise comparison	Cognitive-behavioral therapy vs. Wait list	Problem-solving therapy vs. Wait list
Definition of depression	APAI > 32 21-item BDI > 15 21-item BDI > 10 27-item CDI > 15 CDRS-*R* > 30 DSM-III DSM-III-R DSM-IV	20-item CES-D > 16 27-item CDI > 16 DSM-IV

*APAI* Acholi Psychosocial Assessment Instrument depression symptom scale, *BDI* Beck Depression Inventory, *CES-D* Center for Epidemiologic Study Depression Scale, *CDI* Children’s Depression Inventory, *CDRS-R* Children’s Depression Rating Scale-Revised [10]

#### Were results consistent between direct and indirect comparisons?

Whenever a closed loop is present (Fig. 1.2, and Table 6) there is a possibility that the available direct and indirect comparisons will yield very different effect estimates, a condition we refer to as incoherence, or “inconsistency” used by other authorities [26, 27, 43, 53, 54]. Incoherence can arise for reasons similar to those that can explain heterogeneity and intransitivity (Table 4).

**Table 6:** Glossary of terms

*Certainty:* Quality of the evidence or confidence in the evidence.	
*Direct estimates:* Effect estimate determined from a head-to-head comparison (such as study of A versus B).	
*Indirect estimates:* Effect estimate determined from two or more head-to-head comparisons through a common (such as the relative effect of A versus B by comparing the effect of A versus C and B versus C).	
*Network (multiple-treatment comparisons or multiple-treatment meta-analysis)* ***:*** Effect estimate determined for a particular comparison from the combination of direct and indirect effect estimates.	
*Loop:* A loop of evidence exists when 2 or more direct comparisons contribute to an indirect estimate (e.g., A-B and A-C, contribute to indirect B-C) this loop is considered closed if direct evidence exists between B-C, and open when this direct evidence does not exists.	
*Indirectness:* Term used in *direct evidence* to describe the presence of systematic clinical or methodological differences between head-to-head studies that can act as effect modifiers. These can be in different patients characteristics, ways of administering the interventions, measuring outcomes, or ROB.	
*Intransitivity:* Term used in *indirect evidence* to describe the presence of systematic clinical or methodological differences between head-to-head studies that can act as effect modifiers. These can be in different patients characteristics, ways of administering the interventions, measuring outcomes, or ROB.	
*Heterogeneity* (Inconsistency)*:* The presence of differences in effect estimates between head-to-head studies that assessed the same comparison.	
*Incoherence:* The presence of differences in effect estimates between direct and indirect evidence.	

One can assess incoherence through inspecting the point estimates and the degree of CI or CrI overlap between direct and indirect evidence. In addition, investigators may conduct statistical tests that addresses whether chance can explain difference between direct and indirect comparisons [55, 56]. Unexplained incoherence requires rating down evidence quality.

In the asthma NMA, the direct evidence comparing ICS-L versus leukotriene receptor antagonists (LTRA) suggested a large reduction in exacerbation favoring ICS-L (OR = 0.38; 95%CrI 0.21, 0.68), and the network estimate showed a significant reduction (OR = 0.56, 95%CrI 0.39, 0.76) [12] – from which, one might infer that the indirect estimate showed a substantially smaller effect or, depending on the amount of indirect evidence, none at all. If the authors had provided the indirect estimate and its CrI, one could make the judgment regarding the degree of incoherence. The authors’ statement that they found no incoherence in the network on the basis of statistical tests is somewhat reassuring.

Like conventional meta-analysis, when heterogeneity is high, NMA can use techniques of subgroup analysis and meta-regression to try and explain heterogeneity by identifying modifiers of treatment effects [57, 58]. For example in the NMA addressing adverse events associated with antidepressant medications in children and adolescents [41], the OR for adverse events associated with sertraline use compared to placebo 2.94 (95%CrI 0.94,17.19, I^2^ = 79.3). The authors performed a subgroup analysis based on age and found increased adverse events with sertraline compared to placebo; for children age < 13 years (OR = 12.64, 95%CrI 2.72, 678.43), and in children age > 13 years (OR = 0.59, 95%CrI 0.15, 6.03).

A somewhat less satisfactory way of exploring heterogeneity is to omit studies and determine if the omission influences results. For example, in the mechanical ventilation NMA, the authors examined the robustness of the analysis by excluding 2 studies that included only newborns with gestational age 25–26 weeks [9]. The results showed no changes in the effect estimates.

When direct and indirect evidence vary, and the network estimate is between the two and rated down for incoherence, what estimate is the clinician going to believe? The GRADE approach suggests using the effect estimates from the highest quality evidence, which most commonly will be the direct estimate [31]. Other authorities would argue that, having committed oneself to an NMA, one should always use the network estimates.

For example, the pediatric antidepressants medications NMA included a comparison of Fluoxetine versus Placebo (Table 7) [41]. In this comparison, one can infer from the information presented a rating of the quality of the direct evidence as very low, the indirect evidence as moderate, and the network estimate as very low quality. In this case, following the GRADE approach, the clinician is better off using the effect estimates from the indirect evidence.

**Table 7:** Differences in the evidence certainty across evidence sources in the depression treatment NMA

Comparison	Direct evidence	Direct evidence certainty in estimates	Indirect evidence	Indirect evidence certainty in estimates^f^	Network	Network certainty in estimates
Fluoxetine vs. Placebo	−0.26 (−0.50, −0.03)	⊕OOO VERY LOW^a,b,c^	−1.41 (−2.35, − 0.47)	⊕⊕⊕O MODERATE^d^	− 0.51 (− 0.99, − 0.03)	⊕OOO VERY LOW^b,e^

^a^rated down for RoB

^b^rated down for imprecision (upper CI close to the null)

^c^rated down for heterogeneity (I^2^ = 67.4%)

^d^loops informed the indirect evidence were of low ROB, imprecise (Duloxetine-placebo [SMD = − 0.11 95%CrI -0.3, 0.08; I^2^ = 17%], Duloxetine- Fluoxetine [SMD = − 0.09 95%CrI -0.26, 0.08; I^2^ = 0%], no intransitivity

^e^rated down for incoherence (τ^2^ = 0.33, *P* value = 0.02)

Effect estimates are SMD (95th CI) [41]

^f^Assessed from first order loop Duloxetine-placebo (*n* = 552), Duloxetine- Fluoxetine (*n* = 557), included 7–17 years old children, treated for 10 weeks

#### Is there evidence for publication Bias?

Publication bias results from missing studies [59]. This is because some studies, particularly those with negative results, may never be published. A low risk NMA for publication bias will demonstrate comprehensive search for studies, present symmetrical funnel, and demonstrate insignificant statistical test for publication bias [38]. This assessment requires, however, at least 10 studies. If publication bias is very likely rating down the evidence is warranted.

#### Were treatment ranks presented and were they trustworthy?

Methods are available that allow NMA authors to rank treatments from best to worst [26, 60]. They are often expressed as probabilities that treatments are 1st, 2nd, 3rd etc. best, either in tables (Table 8) or graphically (rankograms). Surface under the ranking (SUCRA) summarizes the information from the rankograms as a single number. Ranking need be made for each outcome –a treatment that is best for one outcome (e.g. benefit) may be worst for another (e.g. harm) [60].

**Table 8:** Asthma treatments strategies effectiveness NMA in improving symptom free days

	1st Rank	2nd Rank	3rd Rank	4th Rank
ICS + LABA	0.95	0.05	0.01	0
ICS low dose	0.02	0.38	0.37	0.24
ICS high dose	0.01	0.33	0.36	0.29
ICS + LTRA	0.02	0.24	0.26	0.45

Ranks are expressed as probabilities that sums to 1. *ICS-L* low-dose inhaled corticosteroids, *ICS-H* medium or high-dose inhaled corticosteroids, *LTRAs* leukotriene receptor antagonists, *LABA*, long-acting b-agonists strategies [12]

Although intuitively appealing, there are a number of reasons why clinicians should not routinely choose a treatment with the higher rankings [61]. First, a treatment that is best in one outcome (e.g., a benefit outcome) may be the worst in another (e.g., a harm outcome). Second, issues such as cost and a clinician’s familiarity with use of a particular treatment may also bear consideration. Third, rankings do not take into account the magnitude of differences in effects between treatments (a first ranked treatment may be only slightly, or a great deal better than the second ranked treatment). Fourth, chance may explain apparent difference between treatments; the use of a measure of uncertainty such as credible intervals for the SUCRA or *p*-value might help to consider the precision of these probabilities [62]. Finally, and most important, the evidence on which rankings are based may be very low quality, and therefore untrustworthy [61].

Although the first ranking may be secure, others are not: the asthma NMA showed that the treatment ranks for 2nd, 3rd, and 4th orders were ICS-L, ICS-H, ICS + LTRA (Table 8) for improving symptom-free days [12]. However, the probability for each treatment were close: 0.38, 0.33, 0.24 respectively, the NMA estimates were imprecise, and of low quality evidence. Therefore, the treatment ranks for the 2nd, 3rd, and 4th orders are untrustworthy.

### Applicability

Just as in conventional systematic reviews and pairwise meta-analysis, applicability may be limited by differences between the clinicial setting and the setting in which the trials were conducted. These limitations may include differences in the patients (e.g. the patient may be younger than those included in the trials); the intervention (e.g. the clinician is considering use of doses differing from those tested in the trials); comparators (e.g. trials used standard care as a comparator, and standard care delivered in the trials differs from standard care in the clinician’s setting); and outcomes (e.g. the clinician is concerned about long-term effects of treatment and trials examined only shorter term outcomes). In any of these situations, the clinician must consider the extent to which trial results apply to their patients and, if such differences exist, potentially refer to other evidence or their own experience in deciding on optimal management.

### Implementation

Returning to the NMA of ventilation modes in preterm infants with RDS [9] (P infants with RDS; I and C; all mechanical ventilation modes; O; mortality), the search strategy included 5 databases, and a grey literature search. Two independent reviewers performed title and abstract screening, full text eligibility, data extraction, and quality assessment, resulting in 20 eligible RCTs, comparing 16 ventilation modes in 2832 infants with gestational age 25–32 weeks (Fig. 2). The authors reported baseline characteristics, and assessed RoB using the Jadad instrument [63]. The authors did not present evidence quality assessment but, as we note in the next paragraph, they present enough information to make this judgment.

All included studies were low RoB. The only NMA estimates for mortality in the entire network in which the CrI did not include HR = 1.0 suggested benefit for time-cycled pressure-limited ventilation (TCPL) (HR = 0.29 95%CrI 0.07, 0.97), HFOV (HR = 0.29 95%CrI 0.08, 0.85), SIMV+VG (HR = 0.12, 95%CrI 0.01, 0.86), and V-C (HR = 0.14 95%CrI 0.02, 0.68) modes compared to SIMV+PSV. Although, the upper CrI of those estimates are close to no difference, you decide to not rate them down for imprecision (refer to the earlier discussion on imprecision). The contributing direct comparisons enrolled similar appropriate patients, the interventions appeared to be administered optimally, and the authors reported no heterogeneity or incoherence. You see little reason why, depending on the direction of results, authors would choose not to submit, or editors to publish these studies, and therefore rate publication bias as undetected. All these comparisons constitute high quality evidence. For every other paired comparison in the network, precision is a major concern.

In the ranking, SIMV+VG mode had the highest probability of being ranked first, though that probability was only 29.7%. The V-C mode had the second highest probability of being ranked first, at 22.8%. Given that there is clear difference between these two modes versus only SIMV+PSV (all other CrI were not precise) the only convincing result is that it is wise to avoid using SIMV+PSV. You therefore conclude that use of TCPL, HFOV, SIMV+VG, or V-C – all of which the pediatrician uses regularly - is reasonable and appropriate.

## Conclusion

NMA is a powerful analytic tool that offers many advantages over a conventional meta-analysis. NMA may, however, be misleading because of a number of problems. First authors may not have not followed the basic standards applicable to any meta-analysis (e.g. comprehensive search, duplicate assessment of eligibility, risk of bias, and data abstraction). Second, trials may suffer limitations in risk of bias, precision, consistency, and indirectness. Third, there may be limitations specific to NMA including intransitivity, incoherence, or uncritical reliance on rankings. Therefore, evaluating the NMA evidence requires serial judgments on the creditability of the process of NMA conduct, and evidence quality assessment. This introductory guide will assist clinicians in their understanding of NMA.

## Abbreviations

A/C: Assist-control ventilation
CI: Confidence or credible interval
CrI: Credible interval
FEV1: Forced expiratory volume in 1 s
GINA: Global initiative for asthma
GRADE: Grading recommendations assessment development and evaluation
HFOV: High-frequency oscillatory ventilation
HR: Hazard ratio
ICS: Inhaled corticosteroids
ICS-H: Medium or high-dose inhaled corticosteroids
ICS-L: Low-dose inhaled corticosteroids
IMV: Intermittent mandatory ventilation
LABA: Long-acting b-agonists strategies
LTRAs: Leukotriene receptor antagonists
MA: Meta-analysis
MD: Mean difference
NMA: Network meta-analysis
OR: Odds ratio
RCT: Randomized controlled trial
RD: Risk difference
RDS: Respiratory distress syndrome
ROB: Risk of bias
RR: Risk ratio
SIMV: Synchronized intermittent mechanical ventilation
SIPPV: Synchronized intermittent positive pressure ventilation
VG: Volume-guarantee ventilation
WL: Wait list
